# A novel cuproptosis-related LncRNA signature: Prognostic and therapeutic value for low grade glioma

**DOI:** 10.3389/fonc.2022.1087762

**Published:** 2023-01-26

**Authors:** Jun Wen, Wenting Zhao, Xiaolei Shu

**Affiliations:** ^1^ Chongqing Cancer Multi-Omics Big Data Application Engineering Research Center, Chongqing University Cancer Hospital, Chongqing, China; ^2^ The First Clinical College, Hubei University of Chinese Medicine, Wuhan, China

**Keywords:** cuproptosis, lncRNA, low grade glioma, The Cancer Genome Atlas, prognosis

## Abstract

**Background:**

As a common primary intracranial tumor, the diagnosis and therapy of low-grade glioma (LGG) remains a pivotal barrier. Cuproptosis, a new way induces cell death, has attracted worldwide attention. However, the relationship between cuproptosis and LGG remains unknown. Our study is all about finding out if there are any genes related to coproptosis that can be used to predict the outcome of LGG.

**Methods:**

RNA data and clinical information were selected from Cancer Genome Atlas (TCGA) datasets and the Genotype-Tissue Expression (GTEx), 5 lncRNAs (GAS5.AS1, MYLK.AS1, AC142472.1, AC011346.1, AL359643.3) were identified by Cox univariate and multivariate regression, as well as LASSO Cox regression. In the training and test sets, a dual validation of the predictive signature comprised of these 5 lncRNAs was undertaken. The findings demonstrate that the risk model is able to predict the survival regression of LGG patients and has a good performance in either the KM curve approach or the ROC curve. GO, GSEA and KEGG were carried out to explore the possible molecular processes that affecting the prognosis of LGG. The characteristics of immune microenvironment were investigated by using CIBERSORT, ESTIMATE and ssGSEA.

**Results:**

We identified five lncRNAs related with cuproptosis that were closely associated with the prognosis of LGG and used these five lncRNAs to develop a risk model. Using this risk model, LGG patients were then divided into high-risk and low-risk groups. The two patient groups had significantly distinct survival characteristics. Analyses of Gene Ontology (GO) and the Kyoto Encyclopedia of Genes and Genomes (KEGG) revealed that the differential genes of the two patient groups were primarily concentrated in neural active ligand-receptor interaction and cytokine-cytokine receptor interaction. The ssGSEA score determined the information related to immune infiltration, and the two groups were differentially expressed in immune subpopulations such as T cells and B cells as well.

**Conclusion:**

Our study discovered 5 cuproptosis-related lncRNAs which contribute to predicting patients’ survival of LGG and provide ideas for the exploration of new targets for LGG in the future.

## 1 Introduction

According to the classification of the World Health Organization, gliomas can be divided into I-IV grades based on the malignant degree of tumor cells, of which grades II-III belong to LGG and grade IV to glioblastoma (1). Glioblastoma is the most frequent malignant intracerebral tumor, accounting for about 57% of all gliomas and 48% of all primary malignant central nervous system tumors (2). Its prognosis is poor, and the median survival time is less than two years (3, 4). With better prognosis, the life expectancy of patients with LGG is often more than 10 years. However, The natural history of these tumors is marked by frequent recurrences, despite the fact that the clinical course of the majority of tumors is initially benign (5). Some patients will ultimately worsen, posing grave risks to human life and health (4).

Since 2016, the World Health Organization (WHO) has added molecular characteristics, such as 1p19q co-deletion, ATRX, TP53, and IDH mutations, in the diagnostic categorization of LGG, offering a more thorough and accurate diagnosis (6, 7). High frequencies of epidermal growth factor receptor (EGFR) amplification (8), TERT promoter mutation (9), and PTEN loss are characteristic in idh wild-type glioblastomas (10). Because the presence of these distinctions impacts the prognosis of LGG, the current therapeutic strategy is deeply influenced by these molecular markers.

Cuproptosis is a unique type of cell death recently discovered (11, 12). Specifically, copper binds directly to the fatty components of the tricarboxylic acid (TCA) cycle, resulting in the accumulation of lipoproteins and the subsequent loss of Fe-S cluster proteins, resulting in protein toxic stress and eventually cell death (13). Recent studies have showed higher levels of copper in lots of malignant tumors compared with normal tissues, such as breast (14), lung (15), colorectal (16), oral (17) and bladder cancers (18). Change of the copper protein levels may contributes to the growth or invasion of tumor (19). Its specific mechanism includes stabilizing the nuclear hypoxia-inducible factor-1 (HIF-1) (19, 20), which provides help to subsequent angiogenesis, and ultimately leads to tumor progression and metastasis.

**Figure 1:**
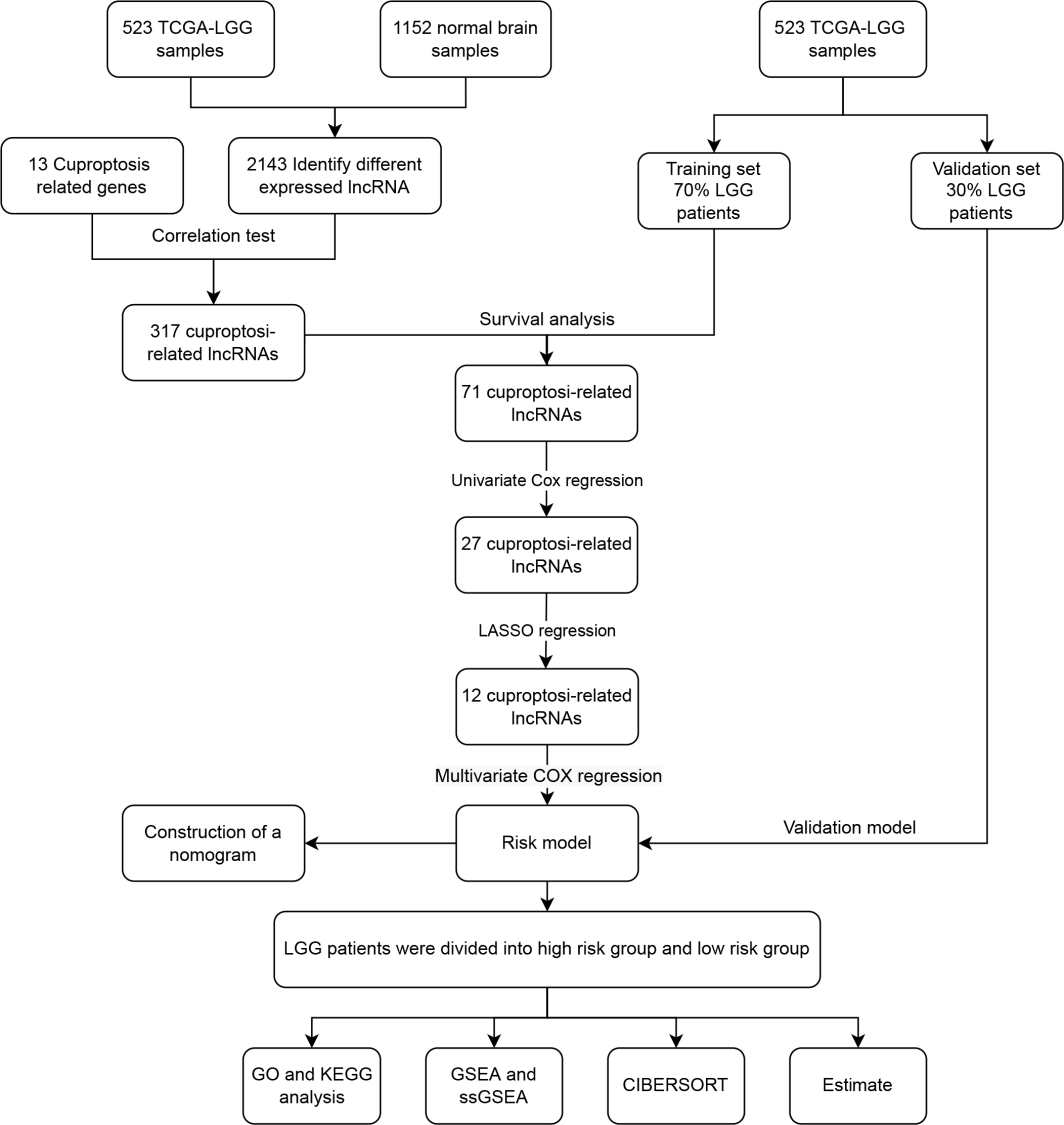
The flow chart of data analysis.

Long non-coding RNA (LncRNA) have a significant role in the control of gene expression and are also implicated in the regulation of programmed cell death (PCD), including autophagy, apoptosis, necrotizing apoptosis, and iron death, which impact the growth of cancer cells in cancer patients (21). In recent years, the lncRNA-constructed LGG prognostic model has demonstrated a degree of success. Shengchao Xu and coworkers developed a model consisting of 19 hypoxia-related lncRNAs that accurately predicts the prognosis and treatment response of LGG patients (22). We developed a model of cuproptosis-related lncRNAs with the purpose of better predicting patient prognosis. **Figure 1** depicts the workflow for this research.

## 2 Materials and methods

### 2.1 Data and resources

The transcriptome profiles and clinical characteristics of LGG patients were retrieved from The Cancer Genome Atlas (TCGA, https://www.tcga.org/) (23), and the transcriptional profiles of normal brain tissues were collected from the Genotype-Tissue Expression Project Database (GTEx, https://commonfund.nih.gov/GTEx). The Counts type data are downloaded from the UCSC xena database (http://xena.ucsc.edu/) (24). Data from patients without complete clinical information were excluded from the study.

### 2.2 Identification of cuproptosis-related lncRNAs

Firstly, the ‘LIMMA’ package (25) in R language (Version 4.1.0) is utilized to pre-process. Then, using the limma software, adjust adj.P values <0.05 and |*logFC*| > 1 condition, identify lncRNAs with differential expression. Using “cuproptosis” as the key word, 13 related genes were selected from PubMed (https://pubmed.ncbi.nlm.nih.gov/). Finally, by the Pearson correlation analysis (26) (with a Correlation coefficient >0.7 and adjust P values <0.001), the cuproptosis-related lncRNA is obtained. Protein-Protein Interaction Networks (PPI, https://cn.string-db.org/) (27) was used to investigate the interaction between these genes and lncRNAs.

### 2.3 Construction of a prognostic cuproptosis-related lncRNA signature

523 LGG patients were randomly selected and divided into training set and test set, in which the training set accounted for 70% and the test set accounted for 30%. According to their median lncRNA expression, patients in the training set were separated into two groups: high and low expressing individuals. When comparing the median survival times of the two groups of patients, we drew KM curves to see whether high or low lncRNA expression had an impact on outcome (28). Univariate COX regression and LASSO regression were used to the KM-curve-selected lncRNAs. The R packages ‘survminer’ and ‘glmnet’ (29) performed the aforementioned tasks. We indicated that univariate and lasso Cox regression analyses were useful in identifying candidate lncRNAs with prognostic significance and reducing the impact of overfitting. Risk signatures were built after a preliminary round of multivariate Cox regression analysis. Risk score= $\sum_{1}^{n}coef_{i}*x_{i}$ (Coefi indicates the correlation coefficient of each ferroptosisrelated signature, and X indicates the level of gene expression) was the formula used to determine the level of danger. The median risk score was used to classify the training and testing sets into high-risk and low-risk groups.

### 2.5 Independent prognostic value of the signature

Then, we analyzed the signature’s predictive power by running univariate and multivariate Cox regressions. The patients’ chances of survival were also estimated using the risk score’s predictive nomogram. The R package “survival” was used to calculate risk scores and determine OS. The model’s accuracy was then assessed with the use of the ROC (constructed using the ‘survminer’ package) and Kaplan-Meier curves (generated by the ‘survivalROC’ package). Dual validation was performed on the training set and the test set to further assess the model’s prediction ability.

### 2.6 Enrichment analysis

Differentially expressed genes (DEGs) were identified between low risk and high risk groups using the limma program in R (with criteria of FDR< 0.05 and | log2 fold change (FC) | ≥1 or greater). Among the many analytical tools available for functional annotation, gene set enrichment analysis (GSEA) stands out as particularly potent. It may be used to decode the expression profile of the whole genome and investigate the connections between various cancer-related, metabolic, transcriptional, and stress-related pathways and activities. Get the HALLMARK genes set from the MSigDB database (https://www.gsea-msigdb.org/GSEA/msigdb) (30), and then run a GSEA analysis using the ‘GSVA’ program (P<0.05 and FDR<0.25) to compare the high-risk and low-risk groups.

To compare the DEGs of high-risk and low-risk groups, we used the R tool ‘ClusterProfiler’ in conjunction with the KEGG and GO databases (31). And infer its purpose from studies of gene sets. Several biological activities and pathways are overrepresented in differentially expressed genes between these two groups; we explore here whether these could contribute to disparities in survival.

### 2.7 Landscape of immune cells infiltration

The “gsva” R package was used for single-sample gene set enrichment analysis (ssGSEA) to assess the immune infiltration status of LGG patients in various risk categories. Using the CIBERSORT software (http://cibersort.stanford.edu/), estimate the cell subgroup abundance by analyzing whole gene expression profiles (32). Scores are produced using the ESTIMATE algorithm (https://bioinformatics.mdanderson.org/public-software/estimate/) to forecast the amount of infiltrating immune and stromal cells, which serve as the foundation for inferring tumor immunity.

### 2.8 RNA extraction and rt-PCR

The U251 glioma cell line and human astrocyte cell line NHA were purchased from Beyotime (Shanghai, China) and cultured in Dulbecco’s Modified Eagle Medium (DMEM; Gibco, NY, USA) containing 10% fetal bovine serum (FBS; Gibco, NY, USA), penicillin (100 units/ml), and streptomycin (100 μg/ml) in a humidified incubator maintained at 5% CO2 and 37° C. Extracted total RNA from cell lines by using Universal RNA Extraction Kit (Takara; Dalian, China). PrimeScript RT-PCR Kit and TB Green were used for reverse transcription and relative lncRNA expression assessment, respectively. Primer information is shown in **Table 1**.

**Table 1:** Primer sequences.

lncRNA	Primer
GAS5-AS1	Forward: 5’-TGTGCCCTTTATACCCACTTT-3’ Reverse: 5’-GCCCAACTAGTGATAGGCATTA-3’
MYLK-AS1	Forward: 5’-TTGCAGTGTTCAGCACTGGCAC-3’ Reverse: 5’-ATTCGACGACCAGTGTTTCAGT-3’
GAPDH	Forward: 5’-GGTGTGAACCATGAGAAGTATGA-3’ Reverse: 5’-GAGTCCTTCCACGATACCAAAG-3’

### 2.9 Statistical analysis

R software version 4.0.4 was used for data analysis. Unpaired Student’s t test and Wilcoxon test were used to compare data conforming to normal distribution and non-normal distribution, respectively. p<0.05 was considered as the threshold for statistical significance.

## 3 Results

### 3.1 Construction of a cuproptosis-related lncRNAs prognostic model signature

**Figure 2:**
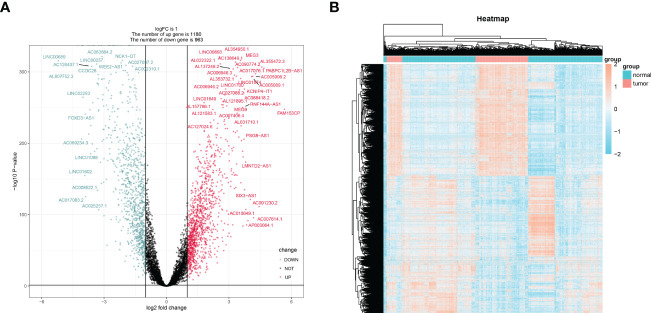
The screening of differentially expressed lncRNAs lncRNAs. The volcano graph **(A)** and heatmap **(B)** showed that 1180 lncRNAs were down-regulated and that 963 lncRNAs were up-regulated in tissues of LGG compared to normal tissues.

Brain tissues from LGG patients and controls showed differential expression for 2143 lncRNAs in TCGA (**Figures 2A, B**). From a search of PubMed, we know that there are 13 genes involved in cuproptosis-related genes: DLST, FDX1, LIAS, SLC31A1, LIPT1, ATP7A, DLD, ATP7B, PDHB, and DBT (33, 34). The chosen genes were used to create a correlation network map with differential expression lncRNAs (**Figure S1**), from which 317 lncRNAs with cuproptosis-related differential expression were extracted. Further confirming the usefulness of these lncRNAs, KM curves were generated for 70% of patients chosen from the TCGA database, and lncRNAs with minor survival significance were omitted. Finally, 71 lncRNAs were successfully extracted.

**Figure 3:**
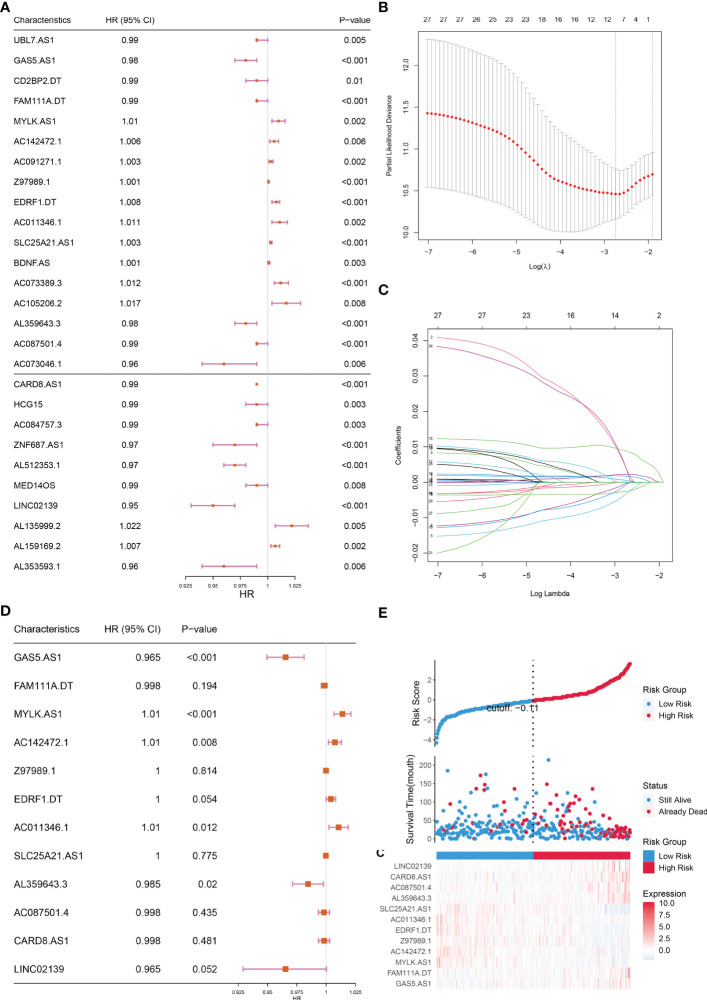
Construction of the prognostic cuproptosis-related lncRNAs signature for in the training set. **(A)** Based on univariate Cox regression analysis, 21 of the 71 cuproptosis-related lncRNAs were screened, as shown by the forest map. **(B, C)** Lasso regression analysis was used to further screen out 12 lncRNAs based on 10-fold cross-validation. **(D)** Forest plot of 12 cuproptosis-related lncRNAs based on Multivariate Cox regression. **(E)** The riskscore distribution, OS, and the Heat map of five lncRNAs of patients in the training set.

The lncRNAs identified in the preceding phase were subjected to univariate and lasso regression analysis. In the univariate regression analysis, 27 lncRNAs were discovered to be substantially related to OS (**Figure 3A**). In addition, LASSO regression analysis of these 27 lncRNAs removed 15 lncRNAs and yielded 12 lncRNAs associated with cuproptosis (**Figures 3B, C**). These 12 lncRNAs underwent multifactorial regression analysis, and a risk model for 5-cuproptosis-related lncRNAs signature was developed (**Figures 3D, E**).

### 3.2 Validation of the prognostic model

**Figure 4:**
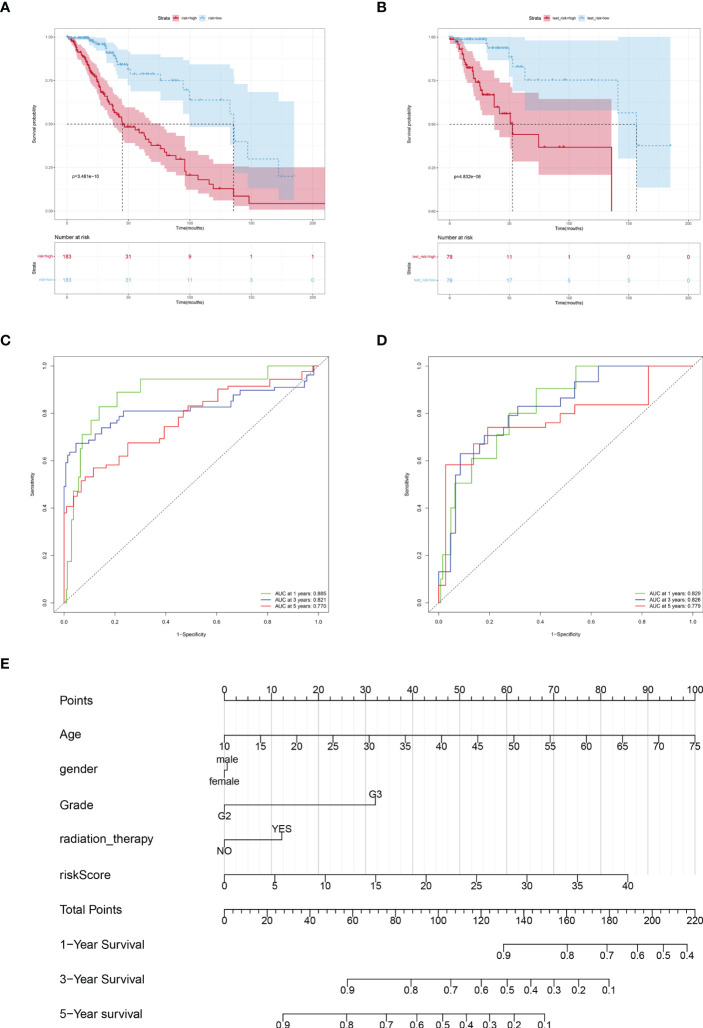
Verification the prognostic value of risk score. Kaplan-Meier curves of LGGs patients in the TCGA training cohort **(A)** and testing cohort **(B)**. AUC values at 1, 3, and 5 years in the TCGA training cohort **(C)** and testing cohort **(D)**. **(E)** Nomogram integrating risk score and clinical variables predicts 1-, 3-, and 5-year OS probabilities.

Based on the risk scores, we plotted KM curves and time-dependent ROC curves (**Figures 4A–D**) for the high-risk and low-risk groups of patients in the training set and the test set (**Figures 4A–D**). As seen in the graph, our model had a high predictive value at 1, 3, and 5 years for both the training and validation sets (AUC were greater than 0.75).

Subsequently, a predictive Nomogram was created, by this 5-cuproptosis-related lncRNA signature (**Figures 4E**). This line graph includes clinical characteristics such as age, gender, and grade. The calibration curve showed that the Nomogram could accurately predict the overall survival at 1, 3, and 5 years (**Figure S2**).

### 3.3 Functional enrichment analysis

**Figure 5:**
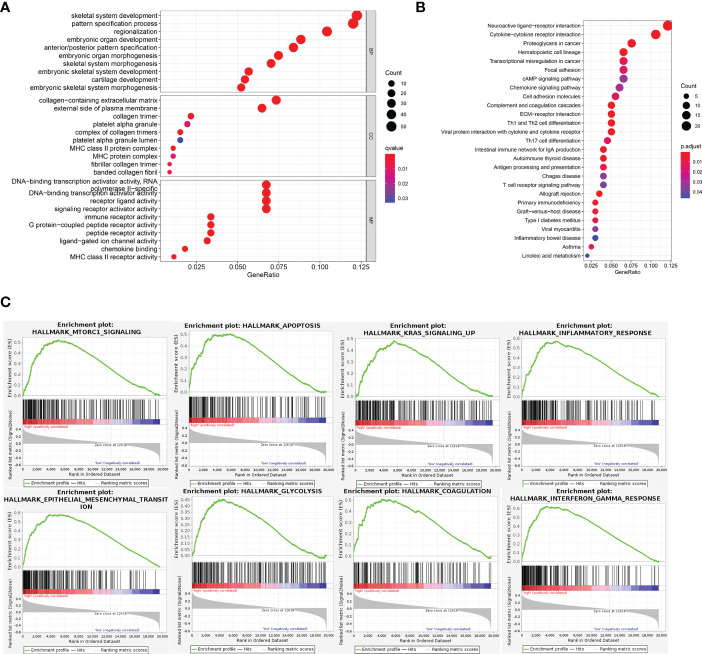
Functional analysis of DEGs. **(A, B)** GO and KEGG pathway enrichment analyses of DEGs in low-risk and high-risk groups. **(C)** In GSEA, the top 8 pathways or biological processes were sorted by P value.

GO enrichment and KEGG pathway were carried out to analysis the possible molecular processes. Results showed that the differential genes were mainly involved in signal pathways such as neuroreceptor-ligand interaction, cytokine-cytokine interaction, and tumor proteoglycan (**Figures 5A, B**). Subsequently GSEA analysis also showed that the differential gene pathway was mainly concentrated in MTORC1 signal pathway and apoptosis, KRAS signal pathway (**Figure 5C**; **Table S1**).

### 3.4 Immune-related analysis of LGG patients

**Figure 6:**
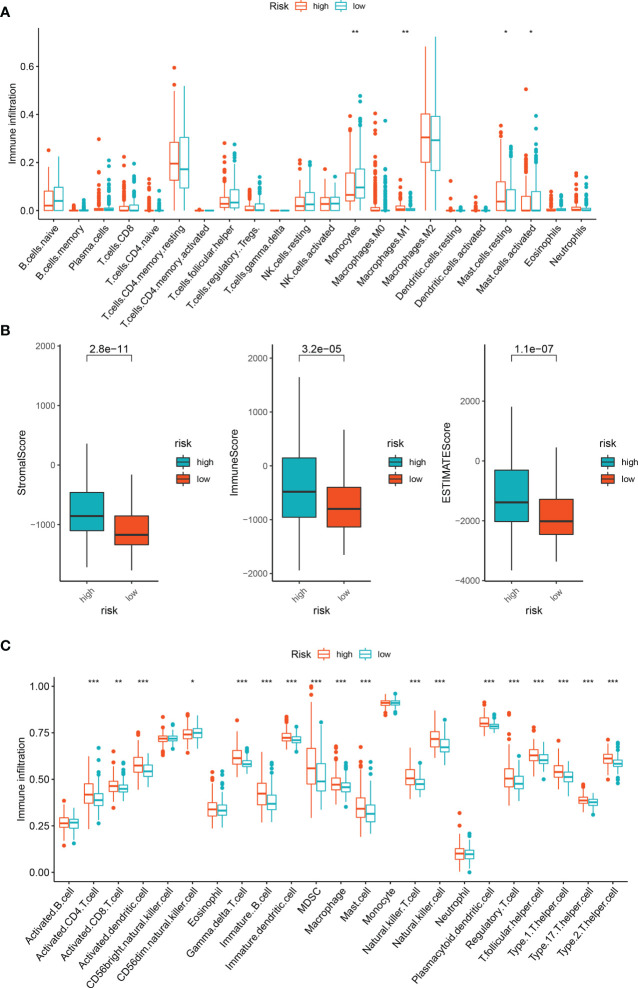
Immune infiltration analysis of DEGs. **(A)** Immune cell subpopulations in ssGSEA. **(B)** Different socres in high- and low-risk group. **(C)** Immune cell subpopulations in CIBERSORT. *p < 0.05, **p < 0.01, and ***p < 0.001.

We employed the CIBERSORT and Estimate method to identify immune cell infiltration in LGG patients, since the enrichment analysis revealed that the association between cuproptosis and LGG is mostly reliant on the tumor inflammatory pathway. We used CIBERSOR and Estimate algorithms to calculate the relative proportion of 22 immune cells in each LGG patient. The correlation analysis between risk score and the level of immune cell infiltration showed that the infiltration degree of many immune cells was different among subgroups (P< 0.05). The results showed that the scores of monocytes and M1 macrophages and mast cells decreased in the high-risk group (**Figure 6**).

### 3.5 rt-PCR was used to verify the expression of lncRNAs in glioma cell line

**Figure 7:**
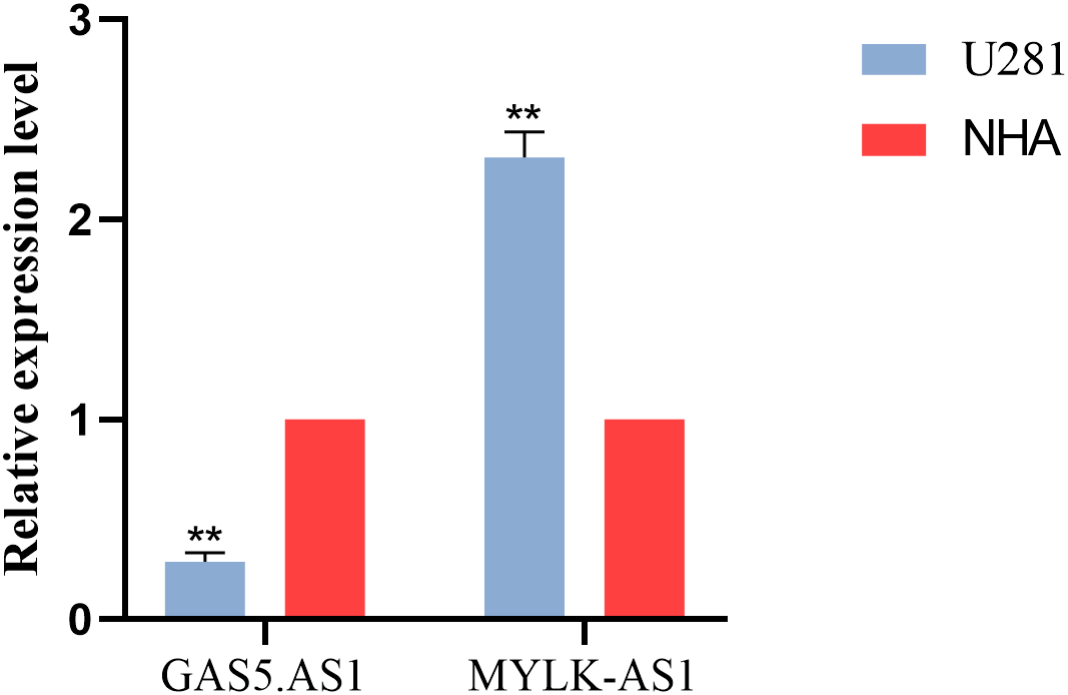
Validation of the expression level of GAS5.AS1 and MYLK.AS1 in cell lines and tissues.

Among the 5 lncRNAs, AC142472.1, AC011346.1, and AL359643.3 lacked relevant studies, therefore MYLK.AS1 and GAS5.AS1, which have been shown to be strongly associated with tumors in prior research, were chosen. In the U251 cell line, the expressions of GAS5.AS1 and MYLK.AS1 were up-regulated and down-regulated, respectively (**Figure 7**).

## 4 Discussion

Low-grade gliomas are primary brain tumors that tend to occur in young people. Common treatments include surgery and chemotherapy, accompany with good prognosis and long survival (35). But with our timely treatment, it will seriously affect the quality of life. Therefore, new approaches to LGG diagnosis and treatment are urgently needed.

Has a fundamental effect on biological processes (36), copper can regulates several biological pathways based on external stimulation (37). The copper accumulation is closely related to tumor proliferation and growth, angiogenesis, and metastasis (19, 37).

In this research, by analyzing the clinical data of LGG patients in TCGA and combining it with coproptosis, we constructed 5 (GAS5.AS1, MYLK.AS1, AC142472.1, AC011346.1, AL359643.3) cuproptosis-related lncRNAs prognostic models, analyzed and predicted their clinical prognosis, and found the relationship between them and tumor immunity.The discriminability and precision of the developed lncRNA signatures were validated using Kaplan-Meier survival analysis and area under the curve (AUC). The test set was then used to validate the risk model’s predictive value. The prognostic model performed well in ROC curve analysis, with auc values between 0.88 and 0.77. In addition, the risk score was determined to be an independent risk factor. Thus, the model demonstrated high clinical predictive value. In addition, the signature consists of just five lncRNAs, making it more applicable to clinical applications than previous signatures.

GAS5-AS1 is a down-regulated gene found in glioma tissues and cells. Its high expression can inhibit the proliferation, migration, and invasion of glioma cells. The expression of GAS5-AS1 is related to the tumor grade of glioma and can be used as a new target for the treatment and prognosis prediction of glioma (38). In glioma tissues and cells, lncRNA GAS5-AS1 was repressed, whereas miR-106b-5p was increased. Through the sponge effect, lncRNA GAS5-AS1 may bind miR-106b-5p, therefore promoting the expression of its target gene TUSC2 and inhibiting the growth and spread of glioma (38). In addition, MYLK-AS1 has been found to promote the growth and invasion of hepatocellular carcinoma cells through EGFR/HER2-ERK1/2 signal pathway (39), At the same time, it can also target miR-424-5p/E2F7 axis, activate VEGFR-2 signal pathway, and promote tumor progression and angiogenesis (39), And promote the invasion of nephroblastoma (40). Combined with our research, it may help us to better understand the molecular mechanism of glioma progression. Our research expands the field and provides a reference and direction for their application in cuproptosis and LGG.

Furthermore, based on the 5 lncRNA risk models developed, we estimated the risk scores of LGG patients in the TCGA database and categorized them into high-risk and low-risk groups. Then, Enrichment Analysis was performed. GSEA analysis revealed that the differences between the high-risk and low-risk groups were primarily enriched in the mTORC1 signal, the KARS signal, and the apoptosis. The mTOR pathway is an important regulator of cell survival or proliferation and plays a central role in regulating many basic cellular processes from protein synthesis to autophagy (41). It has been reported that the expression of mTOR pathway is up-regulated in GBM (42). At the same time, mTOR can promote the differentiation and expansion of CD4+ FoxP3+ regulatory T cells and CD8+ memory T cells, and inhibit CD8+ and CD4+ effector T cells (43, 44). This is consistent with our findings in GSEA, but its specific mechanism remains to be further studied, which provides a reference research direction for cuproptosis-related genes to predict the prognosis of LGG gliomas. Complex signaling cascades stimulate RAS, which then activates downstream signaling pathways to regulate a wide variety of cellular functions (45). The KRAS gene, which is part of the RAS gene family, is tied with glioma development and progression (46, 47). KRAS influences the inflammatory milieu of cancer by activating the MAPK and PI3K signaling pathways, which results in the release of additional IL-6/IL-8 cytokines and cancer cell proliferation (48, 49). As for apoptosis, it is inseparable with tumor and almost participates in the whole process of tumor.

Together, these studies support our findings, while there are still many important questions remain unanswered. The specific mechanism of coproptosis-related lncRNAs in LGG, and how they affect tumor development by affecting immunity need more details.

Our data provides a direction and a certain possibility for the treatment of LGG. But there are still certain limitations. Our sample was based entirely on public databases with limited clinical evidence. The prognostic model established in this study needs further experimental verification.

## Data availability statement

The datasets presented in this study can be found in online repositories. The names of the repository/repositories and accession number(s) can be found in the article/**Supplementary Material**.

## Author contributions

JW conceived the research and prepared the publication, whilst WZ conducted the bioinformatics-related analysis. WZ carried out the experimental validation phase of the research. The study was evaluated and arranged by XS. All authors contributed to editorial changes in the manuscript. All authors read and approved the final manuscript.

## Supplementary material

Supplementary Figure 1 PPI network between cuproptosis-related genes and LGG lncRNAs.

Supplementary Figure 2 Kaplan-Meier curves of OS in 12 DEGs.

Supplementary Table 1 GSEA analysis of DEGs based on HALLMARK genes set.

## References

[1] Louis DN Perry A Wesseling P Brat DJ Cree IA Figarella-Branger D. The 2021 WHO classification of tumors of the central nervous system: a summary. Neuro Oncol (2021) 23:1231–51. doi: 10.1093/neuonc/noab106 PMC8328013 34185076

[2] Ostrom QT Gittleman H Liao P Vecchione-Koval T Wolinsky Y Kruchko C. CBTRUS statistical report: Primary brain and other central nervous system tumors diagnosed in the united states in 2010-2014. Neuro Oncol (2017) 19:v1–v88. doi: 10.1093/neuonc/nox158 29117289 PMC5693142

[3] Ohgaki H Kleihues P. Genetic alterations and signaling pathways in the evolution of gliomas. Cancer Sci (2009) 100:2235–41. doi: 10.1111/j.1349-7006.2009.01308.x PMC11159448 19737147

[4] Tan AC Ashley DM López GY Malinzak M Friedman HS Khasraw M. Management of glioblastoma: State of the art and future directions. CA Cancer J Clin (2020) 70:299–312. doi: 10.3322/caac.21613 32478924

[5] Yu Y Villanueva-Meyer J Grimmer MR Hilz S Solomon DA Choi S. Temozolomide-induced hypermutation is associated with distant recurrence and reduced survival after high-grade transformation of low-grade IDH-mutant gliomas. Neuro Oncol (2021) 23:1872–84. doi: 10.1093/neuonc/noab081 PMC8563321 33823014

[6] Louis DN Perry A Reifenberger G von Deimling A Figarella-Branger D Cavenee WK. The 2016 world health organization classification of tumors of the central nervous system: a summary. Acta Neuropathol (2016) 131:803–20. doi: 10.1007/s00401-016-1545-1 27157931

[7] Ohgaki H Kleihues P. The definition of primary and secondary glioblastoma. Clin Cancer Res (2013) 19:764–72. doi: 10.1158/1078-0432.CCR-12-3002 23209033

[8] Verhaak RGW Hoadley KA Purdom E Wang V Qi Y Wilkerson MD. Integrated genomic analysis identifies clinically relevant subtypes of glioblastoma characterized by abnormalities in PDGFRA, IDH1, EGFR, and NF1. Cancer Cell (2010) 17:98–110. doi: 10.1016/j.ccr.2009.12.020 20129251 PMC2818769

[9] Simon M Hosen I Gousias K Rachakonda S Heidenreich B Gessi M. TERT promoter mutations: a novel independent prognostic factor in primary glioblastomas. Neuro Oncol (2015) 17:45–52. doi: 10.1093/neuonc/nou158 25140036 PMC4483052

[10] Li J Yen C Liaw D Podsypanina K Bose S Wang SI. PTEN, a putative protein tyrosine phosphatase gene mutated in human brain, breast, and prostate cancer. Science (1997) 275:1943–7. doi: 10.1126/science.275.5308.1943 9072974

[11] Kahlson MA Dixon SJ. Copper-induced cell death. Science (2022) 375:1231–2. doi: 10.1126/science.abo3959 35298241

[12] Tang D Chen X Kroemer G. Cuproptosis: a copper-triggered modality of mitochondrial cell death. Cell Res (2022) 32:417–8. doi: 10.1038/s41422-022-00653-7 PMC9061796 35354936

[13] Tsvetkov P Coy S Petrova B Dreishpoon M Verma A Abdusamad M. Copper induces cell death by targeting lipoylated TCA cycle proteins. Science (2022) 375:1254–61. doi: 10.1126/science.abf0529 PMC9273333 35298263

[14] Jouybari L Kiani F Islami F Sanagoo A Sayehmiri F Hosnedlova B. Copper concentrations in breast cancer: A systematic review and meta-analysis. Curr Med Chem (2020) 27:6373–83. doi: 10.2174/0929867326666190918120209 31533596

[15] Zhang X Yang Q. Association between serum copper levels and lung cancer risk: A meta-analysis. J Int Med Res (2018) 46:4863–73. doi: 10.1177/0300060518798507 PMC6300955 30296873

[16] Stepien M Jenab M Freisling H Becker N-P Czuban M Tjønneland A. Pre-diagnostic copper and zinc biomarkers and colorectal cancer risk in the European prospective investigation into cancer and nutrition cohort. Carcinogenesis (2017) 38:699–707. doi: 10.1093/carcin/bgx051 28575311

[17] Chen F Wang J Chen J Yan L Hu Z Wu J. Serum copper and zinc levels and the risk of oral cancer: A new insight based on large-scale case-control study. Oral Dis (2019) 25:80–6. doi: 10.1111/odi.12957 30107072

[18] Basu S Singh MK Singh TB Bhartiya SK Singh SP Shukla VK. Heavy and trace metals in carcinoma of the gallbladder. World J Surg (2013) 37:2641–6. doi: 10.1007/s00268-013-2164-9 23942528

[19] Lelièvre P Sancey L Coll J-L Deniaud A Busser B. The multifaceted roles of copper in cancer: A trace metal element with dysregulated metabolism, but also a target or a bullet for therapy. Cancers (Basel) (2020) 12:E3594. doi: 10.3390/cancers12123594 PMC7760327 33271772

[20] Agostinelli E Vianello F Magliulo G Thomas T Thomas TJ. Nanoparticle strategies for cancer therapeutics: Nucleic acids, polyamines, bovine serum amine oxidase and iron oxide nanoparticles (Review). Int J Oncol (2015) 46:5–16. doi: 10.3892/ijo.2014.2706 25333509

[21] Jiang N Zhang X Gu X Li X Shang L. Progress in understanding the role of lncRNA in programmed cell death. Cell Death Discovery (2021) 7:30. doi: 10.1038/s41420-021-00407-1 33558499 PMC7870930

[22] Xu S Tang L Liu Z Luo C Cheng Q. Hypoxia-related lncRNA correlates with prognosis and immune microenvironment in lower-grade glioma. Front Immunol (2021) 12:731048. doi: 10.3389/fimmu.2021.731048 34659218 PMC8514865

[23] Ganini C Amelio I Bertolo R Bove P Buonomo OC Candi E. Global mapping of cancers: The cancer genome atlas and beyond. Mol Oncol (2021) 15:2823–40. doi: 10.1002/1878-0261.13056 PMC8564642 34245122

[24] Wang S Xiong Y Zhao L Gu K Li Y Zhao F. UCSCXenaShiny: An R/CRAN package for interactive analysis of UCSC xena data. Bioinformatics (2021) 38(2):527–9. doi: 10.1093/bioinformatics/btab561 PMC8723150 34323947

[25] Ritchie ME Phipson B Wu D Hu Y Law CW Shi W. Limma powers differential expression analyses for RNA-sequencing and microarray studies. Nucleic Acids Res (2015) 43:e47. doi: 10.1093/nar/gkv007 25605792 PMC4402510

[26] Bishara AJ Hittner JB. Testing the significance of a correlation with nonnormal data: comparison of Pearson, spearman, transformation, and resampling approaches. Psychol Methods (2012) 17:399–417. doi: 10.1037/a0028087 22563845

[27] Szklarczyk D Gable AL Lyon D Junge A Wyder S Huerta-Cepas J. STRING v11: protein-protein association networks with increased coverage, supporting functional discovery in genome-wide experimental datasets. Nucleic Acids Res (2019) 47:D607–13. doi: 10.1093/nar/gky1131 PMC6323986 30476243

[28] Schober P Vetter TR. Kaplan-Meier Curves, log-rank tests, and cox regression for time-to-Event data. Anesth Analg (2021) 132:969–70. doi: 10.1213/ANE.0000000000005358 33723194

[29] Engebretsen S Bohlin J. Statistical predictions with glmnet. Clin Epigenet (2019) 11:123. doi: 10.1186/s13148-019-0730-1 PMC6708235 31443682

[30] Liberzon A Birger C Thorvaldsdóttir H Ghandi M Mesirov JP Tamayo P. The molecular signatures database (MSigDB) hallmark gene set collection. Cell Syst (2015) 1:417–25. doi: 10.1016/j.cels.2015.12.004 PMC4707969 26771021

[31] Yu G Wang L-G Han Y He Q-Y. clusterProfiler: an r package for comparing biological themes among gene clusters. OMICS (2012) 16:284–7. doi: 10.1089/omi.2011.0118 PMC3339379 22455463

[32] Gentles AJ Newman AM Liu CL Bratman SV Feng W Kim D. The prognostic landscape of genes and infiltrating immune cells across human cancers. Nat Med (2015) 21:938–45. doi: 10.1038/nm.3909 PMC4852857 26193342

[33] Zhou C Li C Zheng Y Huang X. Regulation, genomics, and clinical characteristics of cuproptosis regulators in pan-cancer. Front Oncol (2022) 12:934076. doi: 10.3389/fonc.2022.934076 36387247 PMC9647015

[34] Liu H. Pan-cancer profiles of the cuproptosis gene set. Am J Cancer Res (2022) 12:4074–81. doi: 10.21203/rs.3.rs-1716214/v1 PMC9442004 36119826

[35] Wang TJC Mehta MP. Low-grade glioma radiotherapy treatment and trials. Neurosurg Clin N Am (2019) 30:111–8. doi: 10.1016/j.nec.2018.08.008 30470398

[36] Lm R AL Aa E. Role of copper on mitochondrial function and metabolism. Front Mol Biosci (2021) 8:711227. doi: 10.3389/fmolb.2021.711227 34504870 PMC8421569

[37] Li Y. Copper homeostasis: Emerging target for cancer treatment. IUBMB Life (2020) 72:1900–8. doi: 10.1002/iub.2341 32599675

[38] Huang W Shi Y Han B Wang Q Zhang B Qi C. LncRNA GAS5-AS1 inhibits glioma proliferation, migration, and invasion *via* miR-106b-5p/TUSC2 axis. Hum Cell (2020) 33:416–26. doi: 10.1007/s13577-020-00331-z 32072565

[39] Teng F Zhang J-X Chang Q-M Wu X-B Tang W-G Wang J-F. LncRNA MYLK-AS1 facilitates tumor progression and angiogenesis by targeting miR-424-5p/E2F7 axis and activating VEGFR-2 signaling pathway in hepatocellular carcinoma. J Exp Clin Cancer Res (2020) 39:235. doi: 10.1186/s13046-020-01739-z 33168027 PMC7650167

[40] Zhu S Zhang J Gao X Tang X Cui Y Li D. Silencing of long noncoding RNA MYLK-AS1 suppresses nephroblastoma *via* down-regulation of CCNE1 through transcription factor TCF7L2. J Cell Physiol (2021) 236:5757–70. doi: 10.1002/jcp.30259 33438217

[41] Saxton RA Sabatini DM. mTOR signaling in growth, metabolism, and disease. Cell (2017) 169:361–71. doi: 10.1016/j.cell.2017.03.035 28388417

[42] Dumas AA Pomella N Rosser G Guglielmi L Vinel C Millner TO. Microglia promote glioblastoma *via* mTOR-mediated immunosuppression of the tumour microenvironment. EMBO J (2020) 39:e103790. doi: 10.15252/embj.2019103790 32567735 PMC7396846

[43] Araki K Turner AP Shaffer VO Gangappa S Keller SA Bachmann MF. mTOR regulates memory CD8 T-cell differentiation. Nature (2009) 460:108–12. doi: 10.1038/nature08155 PMC2710807 19543266

[44] Haxhinasto S Mathis D Benoist C. The AKT-mTOR axis regulates *de novo* differentiation of CD4+Foxp3+ cells. J Exp Med (2008) 205:565–74. doi: 10.1084/jem.20071477 PMC2275380 18283119

[45] Mukhopadhyay S Vander Heiden MG McCormick F. The metabolic landscape of RAS-driven cancers from biology to therapy. Nat Cancer (2021) 2:271–83. doi: 10.1038/s43018-021-00184-x PMC8045781 33870211

[46] Guan Q Yuan L Lin A Lin H Huang X Ruan J. KRAS gene polymorphisms are associated with the risk of glioma: a two-center case-control study. Transl Pediatr (2021) 10:579–86. doi: 10.21037/tp-20-359 PMC8039792 33850816

[47] Chiang J Li X Liu APY Qaddoumi I Acharya S Ellison DW. Tectal glioma harbors high rates of KRAS G12R and concomitant KRAS and BRAF alterations. Acta Neuropathol (2020) 139:601–2. doi: 10.1007/s00401-019-02112-x PMC7725430 31822998

[48] Ryall S Zapotocky M Fukuoka K Nobre L Guerreiro Stucklin A Bennett J. Integrated molecular and clinical analysis of 1,000 pediatric low-grade gliomas. Cancer Cell (2020) 37:569–83.e5. doi: 10.1016/j.ccell.2020.03.011 PMC7169997 32289278

[49] Munoz L Yeung YT Grewal T. Oncogenic ras modulates p38 MAPK-mediated inflammatory cytokine production in glioblastoma cells. Cancer Biol Ther (2016) 17:355–63. doi: 10.1080/15384047.2016.1139249 PMC4910925 26794430

