# Supplementary figures and images for: A novel cuproptosis-related LncRNA signature: Prognostic and therapeutic value for low grade glioma
Source: Front Oncol. 2023 Jan 26;12:1087762. doi: 10.3389/fonc.2022.1087762 (PMC9909527; doi:10.3389/fonc.2022.1087762)

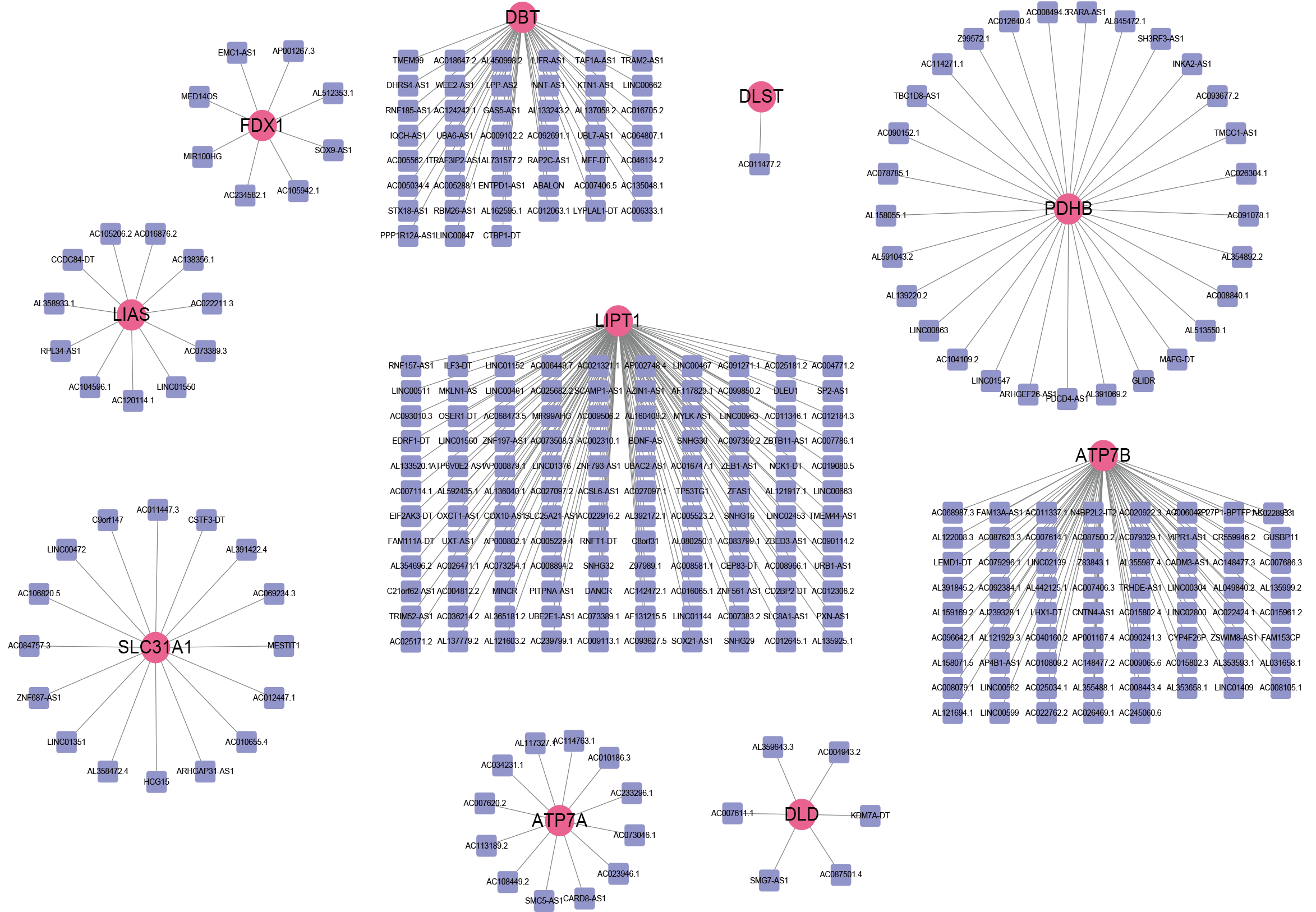

Supplement: Supplementary Figure 1 — PPI network between cuproptosis-related genes and LGG lncRNAs. [file Image_1.tif]

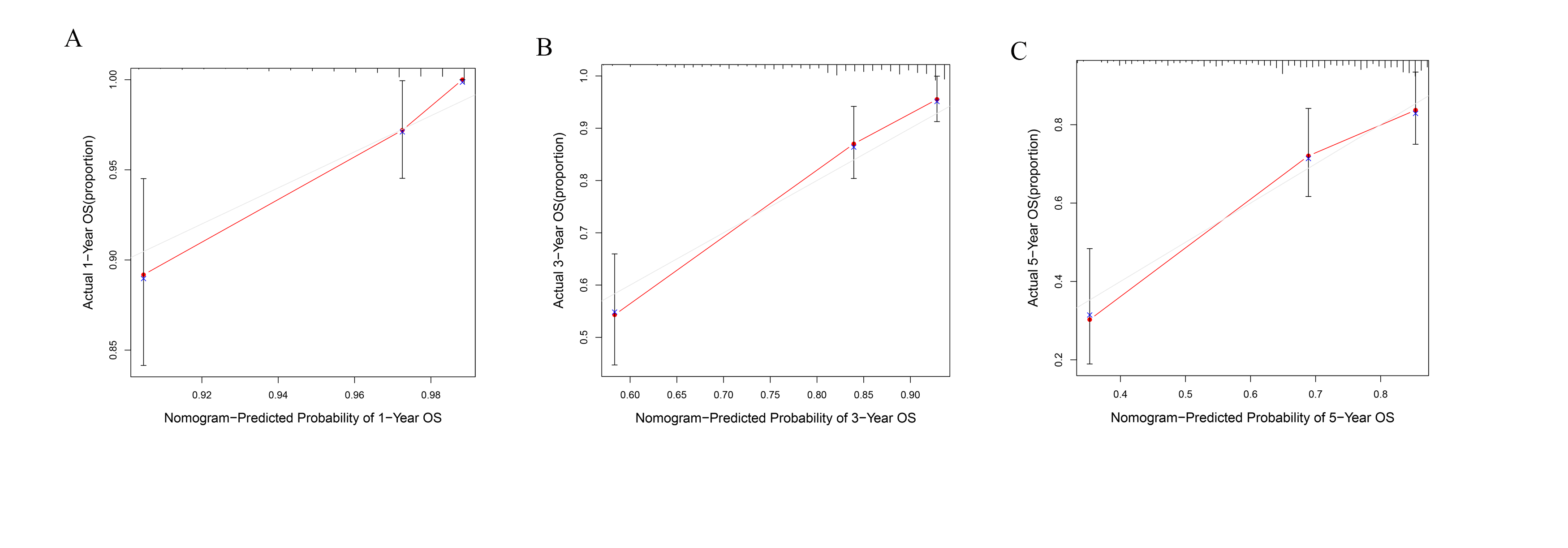

Supplement: Supplementary Figure 2 — Kaplan-Meier curves of OS in 12 DEGs. [file Image_2.tif]
