# Supplementary material for: A novel cuproptosis-related LncRNA signature: Prognostic and therapeutic value for low grade glioma
Source: Front Oncol. 2023 Jan 26;12:1087762. doi: 10.3389/fonc.2022.1087762 (PMC9909527; doi:10.3389/fonc.2022.1087762)
Supplement: Supplementary Table 1 — GSEA analysis of DEGs based on HALLMARK genes set. [file Table_1.docx]

**Table S1**

|  | DETAILS | ES | NES | NOM p-val | FDR q-val |
| --- | --- | --- | --- | --- | --- |
| 1 | HALLMARK_MTORC1_SIGNALING | 200 | 0.52 | 1.8 | 0 |
| 2 | HALLMARK_APOPTOSIS | 160 | 0.5 | 1.79 | 0.002 |
| 3 | HALLMARK_KRAS_SIGNALING_UP | 200 | 0.48 | 1.75 | 0.006 |
| 4 | HALLMARK_INFLAMMATORY_RESPONSE | 199 | 0.57 | 1.75 | 0.01 |
| 5 | HALLMARK_EPITHELIAL_MESENCHYMAL_TRANSITION | 199 | 0.58 | 1.74 | 0.016 |
| 6 | HALLMARK_GLYCOLYSIS | 198 | 0.45 | 1.74 | 0.006 |
| 7 | HALLMARK_COAGULATION | 138 | 0.51 | 1.73 | 0.022 |
| 8 | HALLMARK_IL2_STAT5_SIGNALING | 199 | 0.49 | 1.73 | 0.012 |
| 9 | HALLMARK_COMPLEMENT | 200 | 0.51 | 1.72 | 0.02 |
| 10 | HALLMARK_INTERFERON_GAMMA_RESPONSE | 200 | 0.62 | 1.71 | 0.04 |
| 11 | HALLMARK_APICAL_SURFACE | 44 | 0.47 | 1.7 | 0.011 |
| 12 | HALLMARK_ALLOGRAFT_REJECTION | 200 | 0.58 | 1.7 | 0.048 |
| 13 | HALLMARK_IL6_JAK_STAT3_SIGNALING | 87 | 0.59 | 1.66 | 0.046 |
| 14 | HALLMARK_PI3K_AKT_MTOR_SIGNALING | 104 | 0.48 | 1.65 | 0.006 |
| 15 | HALLMARK_PROTEIN_SECRETION | 96 | 0.55 | 1.64 | 0.016 |
| 16 | HALLMARK_ANGIOGENESIS | 35 | 0.58 | 1.6 | 0.044 |
| 17 | HALLMARK_ESTROGEN_RESPONSE_LATE | 200 | 0.39 | 1.59 | 0.025 |
| 18 | HALLMARK_UV_RESPONSE_DN | 144 | 0.43 | 1.55 | 0.046 |
| 19 | HALLMARK_INTERFERON_ALPHA_RESPONSE | 97 | 0.6 | 1.54 | 0.131 |
| 20 | HALLMARK_ANDROGEN_RESPONSE | 100 | 0.44 | 1.53 | 0.047 |
